# Understanding pharmaceutical care and nurse prescribing in Spain: A grounded theory approach through healthcare professionals’ views and expectations

**DOI:** 10.1371/journal.pone.0260445

**Published:** 2022-01-24

**Authors:** Manuel Lillo-Crespo, Jorge Riquelme-Galindo, Elyne De Baetselier, Bart Van Rompaey, Tinne Dilles

**Affiliations:** 1 Department of Nursing, Faculty of Health Sciences, University of Alicante, Alicante, Spain; 2 Department of Nursing and Pharmaceutical Care, University of Antwerp, Antwerp, Belgium; Universiteit van Amsterdam, NETHERLANDS

## Abstract

**Background:**

Pharmaceutical care has been implemented and regulated differently across Europe with no consensus among countries in relation with professional competencies and especially on nurse prescribing. Demophac Project funded by the European Commission aims to develop a Pan-European Pharmaceutical Care Model with collaboration of 14 partner teams across Europe including Spain where nurse prescribing is starting its implementation at regional level. The aim of the study was to increase understanding of the role of nurses in Pharmaceutical care in Spain after the Nurse Prescribing Regulation approved in 2018 throughout exploring the views and expectations of health professionals involved in the representative settings.

**Methods and findings:**

In depth interviews were conducted in a structure previously agreed by the European Demophac partnership around four topics associated with the Nursing ideal role in pharmaceutical care and the ideal interaction with other healthcare professionals. A grounded-theory approach based on Corbin & Strauss was conducted to interpret collected data from the Spanish most representative settings (primary care, specialized care and residential care for older population). Participants were health professionals involved in pharmaceutical care that accepted to participate (nurses (n = 7), physicians (n = 8) and pharmacists (n = 9)). A pharmaceutical care comprehensive model for the Spanish context considering the recently approved Nurse Prescribing role and the interprofessional collaboration and communication was developed towards facilitating the understanding in such context and the contribution to the unified European Demophac Framework.

**Conclusions:**

Nurses are primarily responsible for population’s Pharmaceutical Care while other professionals pivot on them to provide quality healthcare on a multidisciplinary level. Nurse prescribing may contribute efficiently to the Spanish Health System though more consensus in terms of nurses’ training nationwide and enhancement in communication among different professionals within healthcare organizations is required to achieve adequate integrated care into practice.

## Introduction

Cipolle et al. [1] stated in 2012 that Pharmaceutical care (PC) is the responsible provision of drug therapy for the purpose of achieving definite outcomes that improve the patients’ quality of life. Much attention is paid to PC nowadays worldwide in healthcare contexts due to the increasing interest of Patient Safety Policies. It is a core competency shared by different health professional groups, ranging from medication prescribing to medication administration and its effects’ follow-up. The European picture about PC to date is complex and varied with a lack of consensus. In addition, Nurse Prescribing (NP) has become one of the most controversial competencies among countries and among health professions. The number of Western European and Anglo-Saxon countries where nurses are legally allowed to prescribe medicines is growing [2]. As the prescribing of medicines has traditionally been the task of the medical profession, NP is changing the relationship among different health professions [3]. The countries where nurses can currently prescribe are the United Kingdom, New Zealand [4], Canada, Ireland, France, Sweden and the USA among others [5, 6]. However in those countries the conditions under which nurses prescribe medicines vary considerably, from countries where nurses prescribe independently to countries in which prescribing by nurses is only allowed under strict conditions and the supervision of physicians. For instance, in New Zealand Nurse practitioners are the only category of nurses who can prescribe medicines though their numbers are few compared with the rest of registered nurses in this country [7]. Scientific literature has evidenced the positive effects of NP on medication, as part of PC, and patient outcomes when compared to physician prescribing [8]. However conclusions must remain tentative due to methodological weaknesses of those publications as more randomised controlled designs in the field of NP are required for definitive conclusions about its effects. Moreover NP is just one component of the PC with increasing value at European level. Therefore future studies on NP should be conducted under a unified umbrella of PC, understood as a competency shared by different health professional groups ranging from medication prescribing to medication administration and its effects’ follow-up.

The Demophac Project funded by the European Commission since 2018 aims to develop a Pan-European Pharmaceutical Care Model through the collaboration of 14 partner teams across Europe that could be flexible and inclusive for the different European contexts. Spain is one of the participating nations in Demophac and is experiencing in the last years discussions about PC and NP due to the recent approval of a Royal Law Decree that officially establishes the beginning for its regulation and implementation across the different regions [9]. Recently, the EUPRON Study coordinated by the University of Antwerp in Belgium and developed prior to Demophac in collaboration with 17 satellite European teams from different countries highlighted the mapping of Europe in terms of PC and emphasized the varied NP role across the European regions. EUPRON concluded that NP in collaboration with the other the professionals responsible for PC would have a positive impact on care quality and also that it is necessary to investigate further the perspectives of the professionals involved and reach a consensus at European level [10].

## Background

In Spain, the Royal Decree Law 1302/2018 was published by the end of 2018 regulating the indication, use and authorisation of medicines and health products for human use by nurses [9]. This Royal Decree Law opened the door for the Autonomous Communities’ Governments to specify the skills, responsibilities and tasks that nurses must know and carry out in terms of prescribing, as well as to cover actions not currently having that coverage. Historically in Spain, the role of nurses in PC has been based on the Care Prescribing derived from nursing diagnoses as well as the medication administration though never in relation with legal prescribing of any type of medicine. Only in urgent situations in which the physicians were not present nurses have been assumed to prescribe medications being a legal gap all over the country as it was also highlighted by Romero-Collado et al. [11] in the case of Primary Care Nurses who have routinely prescribed vaccinations, topical antiseptics, antipyretics, wound care dressings and any health care supply including those for diabetes and incontinence though without formal legalization. Nurses indicated that full legalization would increase professional autonomy and contribute positively to the profession, as an example of how policy can have an impact on practice. Until 2018, prescribing had always been associated only to Spanish physicians and pharmacists, also known as facultatives, a term that recognizes them as the professionals with the right to prescribe. In Spain the Nurse Practitioner specialization has never come to light in comparison with other Centre and Northern European countries and consequently a Nurse specialty with a NP recognition in terms of medication has never been developed apart from the basic training on Pharmacology they must achieve during undergraduate studies.

Nowadays in Spain, several thousand experienced registered nurses, educated at Higher Education Institutions since the 70s, meet the education requirements for NP according to other countries’ regulatory bodies where NP has been functioning for years. The Spanish Government has recently published the specific plan for extending specific prescribing responsibilities to registered nurses within the approved Royal Decree Law. The official document specifies that Autonomous Communities (17 in total) must regulate how those responsibilities will be implemented in practice and therefore regulations are needed at a regional level to allow nurses with the appropriate education to be designated prescribers in their named area of specialisation. However, the Ministry of Health has only mentioned some potential examples that should be ideally approached by the 17 Autonomous Communities though none of them have been articulated in practice until now. The examples provided by the Ministry are the ones that have been classified as being in a legal gap such as: vaccination calendars, the use of oxytocin by Midwives in deliveries, medication pre-specified by protocols and guidelines (such as protocols for pain, palliative care and dementia), chronic diseases’ medication follow-up, or the use of therapeutic dressings and other pharmaceutical products.

At this moment, discussions on the type of prescription, the medicines allowed, the required training for nurses with prescribing rights are factors that have not been defined in-depth and need to be clarified to minimise the controversial discussions and impact of other prescribers on the new NP role as part of the PC. The Spanish situation to date is similar to others’ present and past situations in Europe and worldwide. Spanish nurses complete a four-year education programme at higher education institutions with a comparable number of European Credit Transfer (ECT) with countries where NP is allowed. Despite their training the Spanish Nurses have never had the chance to prescribe until now not even with any of the recognized specializations. According to the Spanish General Nursing Council the NP is described as the capacity of the nurse with relation to management, evaluation and provision of nursing care to select, under professional criteria, different materials, products, devices and medication by aiming to satisfy population’s health needs supported by the nursing judgment and administered in terms of care [12]. The first autonomous community in Spain that has adapted the Royal Decree Law is the Balearic Islands followed by Andalucia, Catalonia and the Comunidad Valenciana, where our study took place. In 2008, an article was published highlighting the importance of prescribing by nurses in Spain to improve the quality of care. It described the different types of NP and mentioned professionalism and interdisciplinary work in the field of PC, understanding NP as an improvement and not as a threat to other prescribing professionals [6]. In 2017 Romero-Collado et al. [13] conducted a cross-sectional study exploring course content related to Pharmacology and healthcare products and supplies in all nursing degree programs in Spain. The analysis showed that nurses’ training in Spain during the basic current degree program provides the adequate knowledge and skills needed to prescribe healthcare products or supplies and medications without physicians’ prescription and without the need for additional training. Such study highlighted the importance of Government regulation of nursing education in Spain to be aligned with nursing competencies, curriculum standards, clinical practice and evidence-based research to provide the maximum level of confidence for nursing professionals themselves and their patients.

## The study

### Aim

The purpose of this study was to understand the current role and competencies of nurses in Pharmaceutical care in Spain emphasizing the recently approved Nurse Prescribing Regulation throughout exploring the views and expectations of health professionals involved in the settings where such type of care currently takes place.

### Design

A grounded-theory approach based on Corbin & Strauss method was conducted through in-depth interviews. Such approach is adequate to develop concepts that represent the participants’ experiences and explore complex and latent social processes and patterns (Strauss and Corbin, 2008). Grounded theory is a systematic methodology involving the construction of theories through methodological gathering and analysis of data [14]. Grounded theory was the method of choice because it allows researchers to engage the data to respond actively to a given phenomenon, ultimately generating a theory that offers an action map for a particular situation [15].

### Sample and participants

Twenty-four participants belonging to the health professional groups involved directly in PC in Spain (physicians, nurses and pharmacists) from the most representative settings (Primary Care, Specialized Care and Residential Care for Older Adults) were recruited through purposing sampling and interviewed as it was required at the beginning by Demophac Lead Partner Team. In total: 7 nurses, 8 physicians and 9 pharmacists were selected and all accepted to participate. 15 out of the 24 participants were women and all of them came from clinical practice from the different settings except for one of the nurses who was a manager: 8 came from Specialized Care, 8 from Primary Care and 8 from Residential Care. Data Saturation was reached after 9 interviews. Interviewees provided socio-demographic data before starting the interviews (Table 1). Participants were intended to be representative, being experienced in clinical practice or healthcare management and willing to give their opinion about the topic. All of them were recommended by other professionals, who performed as key informants, following a snowball sampling process. The inclusion criteria were:

- having a minimum of 5 years of experience in their working setting.
- dedicating part of their working time to PC every day.
- being actively working at that moment in tasks related to PC.
- living in Spain and having completed their education in Spain.
- having a background associated to management and administration as well as at least in one of the following fields: clinical practice, research, education and policy.

**Table 1:** Participants’ sociodemographic characteristics.

INTERVIEW	PROFESSION	GENDER	AGE	WORKING ENVIRONMENT	MAIN WORKING FIELD	YEARS OF EXPERIENCE	PHARMACEUTICAL CARE WORK (HOURS PER WEEK)
1	Physician	Female	41	Specialized Care	Clinical Practice	19	40
2	Nurse	Female	50	Specialized Care	Management	25	60
3	Pharmacist	Female	56	Specialized Care	Clinical Practice	25	40
4	Physician	Female	60	Primary Care	Clinical Practice	32	6
5	Nurse	Male	34	Primary Care	Clinical Practice	13	35
6	Pharmacist	Female	30	Primary Care	Clinical Practice	6	40
7	Physician	Female	51	Residential Care	Clinical Practice	25	40
8	Nurse	Female	63	Residential Care	Clinical Practice	43	30
9	Pharmacist	Male	28	Residential Care	Clinical Practice	5	10
10	Physician	Female	32	Specialized Care	Clinical Practice	7	40
11	Physician	Male	43	Specialized Care	Clinical Practice	19	40
12	Pharmacist	Male	48	Specialized Care	Clinical Practice	19	40
13	Pharmacist	Female	35	Specialized Care	Clinical Practice	6	60
14	Nurse	Female	34	Specialized Care	Clinical Practice	6	40
15	Physician	Male	38	Primary Care	Clinical Practice	14	40
16	Physician	Female	40	Primary Care	Clinical Practice	16	40
17	Pharmacist	Male	43	Primary Care	Clinical Practice	14	50
18	Pharmacist	Male	49	Primary Care	Clinical Practice	16	40
19	Nurse	Male	44	Primary Care	Clinical Practice	18	40
20	Physician	Female	38	Residential Care	Clinical Practice	14	60
21	Nurse	Female	36	Residential Care	Clinical Practice	20	40
22	Nurse	Male	40	Residential Care	Clinical Practice	16	40
23	Pharmacist	Female	50	Residential Care	Clinical Practice	20	40
24	Pharmacist	Female	48	Residential Care	Clinical Practice	14	40

Interviewees were firstly contacted via telephone by the principal investigator and once having been informed about the study aims and characteristics and having accepted participation voluntarily, they were required to sign up a written informed consent document. All of them had the chance to abandon the study at any time and decide where to meet for the interviews. In all cases they preferred their working context.

### Data collection

Interviews took place from February to March 2019 lasting 60 minutes approximately and conducted in Spanish, the mother language of all participants. The principal investigator previously had to contact participants’ organizations for permissions. The interviews were audio-recorded with participants’ consent and conducted by two members of the research team: one of them performing as interviewer while the other took notes about the ideas that came out, the non-verbal communication and also controlled the audio-recording. Interviewers had specific training to conduct the qualitative technique selected. Interviewers let participants express their perceptions freely without any manipulation. Interviews consisted of open-ended questions in a structure previously agreed by the European Demophac partnership around four topics associated with the Nursing ideal role in PC and the ideal interaction with other healthcare professionals finishing with a classic SWOT matrix which is a strategic planning technique used to identify strengths, weaknesses, opportunities, and threats. The main four topics were: (a) Nursing responsibilities; (b) Tasks related to those responsibilities; (c) Nursing Interaction with physicians and pharmacists; (d) SWOT analysis about the current situation in Spain [16]. The interviews were part of the Demophac study [17] although those questions directly asking about NP in Spain were specifically context-based for the Spanish team due to the historical transitioning situation in the country. Once each interview had finished the interviewer contrasted the data collected with respondents.

### Ethical consideration

The Ethical Advisory Committee on Social and Human Sciences of the University of Antwerp in Belgium approved to start collecting data in Demophac study on 30 October 2019. Consents from the selected organizations’ chiefs and also from participants were collected and kept assuring the principles of privacy and data protection.

### Data analysis

According to Corbin and Strauss, the theory starts from the study data after a systematic analysis of such data. Its purpose is to make the theory as close as possible to reality based on experience, minimizing speculation [18]. We chose this methodology in order to extract a comprehensive theoretical model and key concepts about PC and NP that could contribute to understand the phenomena in Spain, guide the practice and prospectively provide feedback to the Demophac consensus Model. The analysis procedure was performed according to Gale et al. [19] by 3 experienced researchers (two of them were the interviewers and the third one did not have contact with participants) producing an analysis triangulation. After the interviews’ transcription [20], central categories and connecting categories were extracted following the steps established by the grounded theory analysis method. Software tools for analysis were not used. Firstly, through open coding, the main codes were extracted and added to a codebook (Table 2) performing line-by-line analysis and identifying those codes in addition to their dimensions. Secondly, axial coding was carried out to establish the relationship of the central categories with the connecting categories. Finally, on reaching theoretical saturation, selective coding was used to integrate and refine the theory [14]. Once the answers had been classified, we draw conclusions contrasting between the views of the professionals, their professional backgrounds and work contexts.

**Table 2:** Data analysis codebook.

CODES	DIMENSIONS	CENTRAL CATEGORIES	CONNECTING CATEGORIES (Recurrent in all the central categories)
Pharmaceutical Care	Pharmaceutical care description (holistic view)	1	-
Nurses and Pharmaceutical Care	Holistic view of the nurses’ role in Pharmaceutical Care	1	-
Nurses’ tasks	Nurses’ tasks in Pharmaceutical Care	2	-
Nurses’ responsibilities	Nurses’ responsibilities in Pharmaceutical Care	2	-
Nurse prescribing	Nurse prescribing description (holistic view)	-	1
Complementary Collaborative Prescribing	Delegated, semi-autonomous prescribing	-	1
Protocolised Collaborative Prescribing	Standardised prescribing	-	1
Independent Prescribing	Autonomous prescribing	-	1
NP Benefits	Nurse prescribing benefits	-	1
NP Drawbacks	Nurse prescribing drawbacks	-	1
PC Collaboration	Interprofessional collaboration	-	2
PC Communication	Interprofessional communication	-	2

### Rigour and reliability

In order to guarantee the scientific rigour of the results, the following interventions were carried out in the interviews [21]:

- The interviewer introduced himself as a “researcher” without revealing its professional group and had no previous professional or personal relationship with the interviewee.
- The interviewers always kept a neutral behaviour especially in terms of non-verbal communication by taking care of never expressing emotions or attitudes towards interviewees’ responses.
- Questions were formulated neutrally and openly.
- During one research team meeting, a glossary of terms was developed in order to scientifically define critical words used along the interviews supporting the interviewer this way.
- Interviews were transcribed word by word to maintain the specificity of the answers.
- Participants were informed back about their answers and had the chance to review the transcriptions and transmit their feedback.
- Later a translation and back-translation of the Spanish quotes into English were performed towards this publication.

## Findings

**Fig 1:**
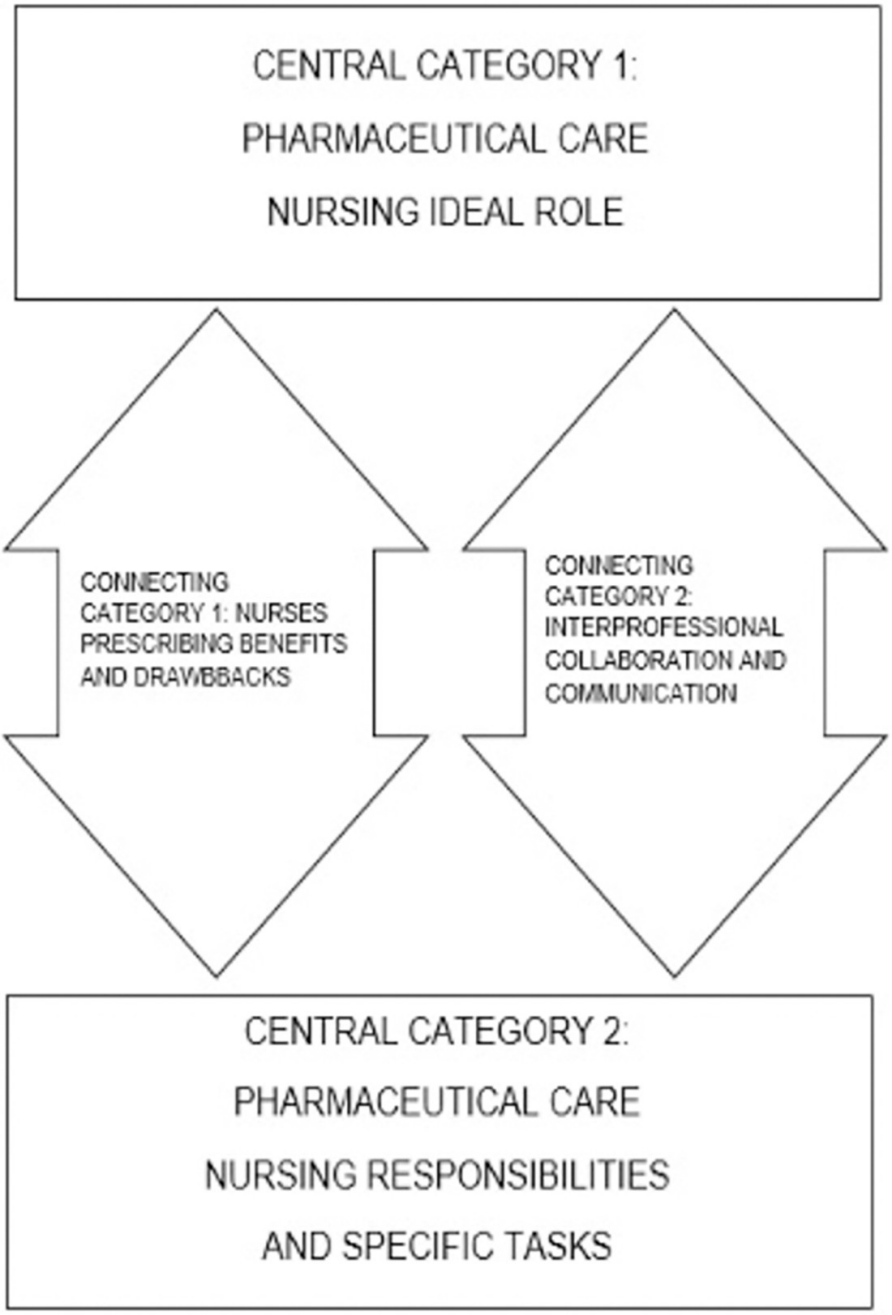
Comprehensive model about pharmaceutical care in Spain.

Our results outlined a better understanding of what comprises the PC in Spain in a historical moment of change. Even though the interviews mainly focused on PC according to Demophac Project aims, due to the historical situation lived about NP in Spain at that moment most of the participants emphasized on such topic in all their responses becoming a recurrent content. Our comprehensive theoretical model is an action map for the understanding of the phenomenon in one specific context. Two central or supporting categories were identified in relation with the codes extracted from the interviews: PC Nursing ideal role and Nursing responsibilities and tasks. Moreover, the theoretical model that emerged from these data highlights that nurses are primarily responsible for population’s Pharmaceutical Care while other professionals pivot on them to provide quality healthcare on a multidisciplinary level and depicts the interrelatedness of the two central categories throughout other two transversal or connecting ones, both describing the role of nurses within PC and its interactions with other professionals. Fig 1 represents the comprehensive theoretical model that we reached for the phenomenon understanding. It describes how the connecting category related to NP is crucial towards understanding PC in Spain at this transitioning historical moment as well as the one about interprofessional communication and collaboration; and also how both are the connecting pillars with the other two central ones. However as there was not a consensus in participants’ responses about the form in which NP and interprofessional relation should and will be implemented our model is flexible and could be adapted to different contexts. It outlines the key strategies, represented in the connecting categories, that should be further developed towards shaping the PC Nursing ideal role in Spain and the Nursing PC responsibilities and specific tasks. It is understood that NP plays a crucial part as well as the collaboration and communication among different professional groups for the PC nursing ideal role and also for the nursing responsibilities and specific tasks. Our model is flexible and inclusive for different contexts in Spain as they could be approached from distinct perspectives according to the Autonomous Community and adapted to the current situation whichever the way of NP and interprofessional collaboration is implemented.

The rationale and major assumptions of this comprehensive model are:

### Central category 1: PC nursing ideal role

According to interviewees the nurses’ role in PC is characterised by its closeness to patients and its coordination role among the rest of health professionals. Nurses take care of patient’s needs, observe patient’s ongoing, communicate with pharmacists in relation to treatment’s revision and administer such treatment prescribed by physicians. Participants emphasized that Nurses are the pivotal professionals to provide quality care to patients:

*“The nurse is not only administering the treatment*, *she is also controlling whether the patient is taking it or not*, *if that is the right treatment for the patient*, *if the patient has any problem with the treatment…*”.

P1

*“Without nurses*, *we wouldn’t have such efficient pharmaceutical care*. *They must be the first and last filter of medication and care”*.

P3

Participants reported that nurses control patients’ medication follow-up in relation with their individual situation and therefore providing person-centred PC. Among all health professionals patients trust nurses the most to tell them what is happening to them, their concerns about their health problems and medication:

“*…by being near the patient*…*by doing the patients’ follow-up…patients feel very comfortable talking to the nurse…it is all about empathy and compassion”*

P4

Nurses were considered as independent professionals in terms of care, and they are used to work in multidisciplinary teams. However, nurses experience and knowledge about drugs are not as developed as other professional groups due to their training. Moreover, they consider themselves as a strategic link with patients and families as well as the last step in the PC process:

“*(The nurse) is the one who is permanently helping the physician with drugs*”

P6

*“Nurse’s office is usually next-door physician’s office*…*because they are complementary in terms of PC”*

P7

### Central category 2: Nursing responsibilities and specific tasks

During the interviews nurses were recognised as responsible agents for medication administration, patients’ follow-up and adherence to treatments as well as for monitoring the adverse effects and reporting medication errors:

*“Nurses do the last checking and evaluate that the PC tasks taken by the physician and pharmacist are correct while maintaining patient’s safety*…*they confirm that the pill is the right one*, *that it corresponds to the right patient…the administration is done through the right way*, *which is extremely important…besides they report any adverse effect or any interactions to the rest of the team so that they can make decisions properly*. *They must be the endpoint of any treatment”*

P9

It was taken for granted by all participants during the interviews that Nurses are autonomous for NP in several situations accepted by the Spanish society though not legally in which physician prescription is not possible such as: the use of drugs in critical situations, during deliveries by midwives, according to protocols and guidelines related to vaccinations, pain management, palliative care and neurological disorders in chronic situations such as dementia.

*“Their responsibility is different according to the route of administration [*…*] and the situation…they are perfectly aware of all the routes and know how to act when an urgent situation happens…”*

P10

Nurses were also identified as the key providers of patient education in terms of cares and medication:

*“…they strengthen information about the medication*, *indicate adverse effects and strengthen adherence to treatment*…*”*

P11

During the past years there has been a growth in the old dependent population in Spain, directly affecting the Nursing competencies regarding PC. One of the most prevalent situations is related with neurodegenerative diseases such as Alzheimer disease and other dementias usually associated with old adults. These people have been traditionally cared at home though recently are transitioning to live and be cared at healthcare facilities where nurses’ role is changing towards making continuing decisions about medication in order to manage symptoms associated with agitation, confusion, sleep disorders, delirium or aggressiveness. Professionals interviewed especially those from the residential care settings highlighted the importance of the nursing professionals focusing on specific cares for Dementia people ranging from non-pharmacological initiatives to medication protocols.

*“…the medications that we mostly use are antipsychotics*. *We know which the adequate dose is in order to manage the patient’s symptoms and we also know the associated complications and how those drugs affect them…”*

P2

*“…our aim is to keep Alzheimer’s Disease people as active as possible and therefore we cognitively stimulate them along the day*. *Thus*, *we need to use music therapy*, *play board games and practice sport to avoid as much as possible the use of drugs to manage symptoms…though sometimes all the non-pharmacological therapies fail and we need to apply the medication protocols…”*

P5

*“…person centred care is crucial for these people as they react differently to the same therapy compared with other patients*. *We need to explore their wishes and needs in order to adapt medication and non-drug therapy to keep them as active as possible…”*

P8

### Connecting category 1: Nurse prescribing benefits and drawbacks

Disagreements were reported by some physicians and pharmacists about NP as such concept does not seem to be understood equally by everyone and it does not belong to the Spanish tradition within the Health System that the nurses could prescribe independently. Most of the physicians and pharmacists tended to think directly about independent NP when the interviewer referred only to NP in the interviews and therefore the concept had to be clarified. This may be related to the political situation in Spain where the NP decree law has been approved recently and the implementation is advancing differently in the 17 regions. Most of the interviewees including nurses discussed about the lack of specific training in NP and discrepancies associated with the type of NP came out ranging from delegated to independent forms. Therefore, there was no consensus in deciding to what extent nurses could prescribe, where the limit is and what it would entail even though there were no voices among participants claiming for a totally dependency in NP:

*“The nurse prescription has supposed a “civil war” among us*. *Nobody knows what the Royal Decree Law is about and not even the nursing colleagues agree*. *In the end we do what we’re allowed to do and not what we’re capable for”*

P14

“*In my opinion*, *the prescription belongs to the physicians*”

P15

*“Nurses should struggle with their limits*…*they must establish the nurse prescribing limits”*

P16

*“They may have a lot of experience in care and self-care*, *administration routes…but in pharmaceutical care and concretely referring to prescribing*, *they need more conceptual basis”*

P17

“*They should get trained much more in Pharmacology because they do not have enough knowledge for prescribing*”

P18

Different types of NP were discussed. In fact, all participants referred to a model of collaborative prescribing at different levels in PC by using different terms such as: delegated, semi-autonomous, complementary and collaborative prescribing; understood as the type of NP in which the nurse adjusts the doses, taking into account how the patient evolves, and directly collaborating with other professionals:

“*Making decisions about drug doses should be a collaborative prescribing*”

P20

*“Prescribing implies an important collaboration among all professionals*…*if not gaps may arise…”*

P23

*“Collaborative prescribing on major diseases would be ideal*. *If you as a nurse have more pharmacological information and also report that information to the pharmacist and physician*, *the patients’ safety would be highly ensured […]”*

P15

The standardised or protocolised prescribing also emerged during interviews in which participants use clinical practice guidelines as a basis for acting in emergency situations or current situations which have been established under an algorithm, guideline, protocol or clinical pathway (for instance in CPR, critical patients, palliative patients, etc.):

*“Prescription based on protocols and guidelines is referred by the Spanish Royal Decree which implies a training with a deep knowledge of what is being done and according to the needs of the moment*…*the problem is that those protocols have not been developed in all the regions”*

P9

*“With limits it could work*. *There must be some protocols or something that can be performed in accordance with”*

P7

“*Nurses have a lack of training and therefore it could work well if they could do that as long as there are standardised protocols*”

P11

“*I think they should intervene but always in collaboration with the physician*”

P4

*“It is true that in some organizations*…*though not in all…there are protocols and other documents that guide the administration of a certain drug in absence of a physician or without a prescription…and nurses are used to cope with those situations without a legal framework…they have the experience”*

P5

*“They are independent when it comes to administering one drug without having to tell me to prescribe it*, *and then they tell me to prescribe it*…*but this happens in very specific settings with experienced nurses”*

P19

In the collaborative prescribing type, there were discrepancies among the interviewed nurses. Some of them said that they should not act freely either in administering or prescribing without a prior prescription based on the diagnosis. In other words, they maintain that there must be always a medical diagnosis in both cases in order to be able to adapt the initial prescription:

“*I believe that we should not act freely either to prescribe it or to administer it without prior clear prescription*”

P22

A third type of NP that emerged was the totally independent/autonomous prescribing in which both the assessment and the diagnosis as well as the prescription are made by the nurse. Participants referred to this type only in very specific clinical cases:

“*Independent for pain management of course*!”

P19

“*In healing a wound by deciding on wound dressing or antibiotic treatment nurses are the most appropriate professionals for prescribing*”

P11

*“I think it could be a great support nowadays for the health system to have a complete nurse prescribing […] In chronic diseases*, *in wound care*, *in topical issues that are not systemic*…*amongst others*, *such as palliative sedation and dementia people pharmacological interventions*, *for instance during disruptive episodes*, *a nurse could do it perfectly”*

P16

The benefits of implementing NP would be firstly the legal coverage of those practices that have been implemented for a long time and were not recognised for nurses determining a legal gap in Spain. Consequently, patient care would be improved and healthcare costs would be reduced according to interviewees:

“*The role they’re performing without any regulation would be covered*”

P6

*“they save time*, *make improvements in patient care*, *avoid errors*, *avoid communication failures… in other words*, *everything is more under control*, *flowing the right way*, *more effective and therefore efficient”*

P7

“*They evidence the health costs of iatrogenic situations and this may justify to increase of nursing staff within our health system*”

P12

However, all interviewees agreed there should be more specific training in Pharmacology for nurses and continuing professional development. All the professionals interviewed showed that there is a certain sense of unsafety in nurses when it comes to prescribing into practice due to the lack of knowledge of nurses about medication. Training as well as research and multidisciplinary work were the key elements highlighted to improve patient care:

*“Research to improve is necessary in Nursing Education*. *Own research for nurses and Nursing support for physicians and pharmacists are crucial”*

P7

*“It is always the problem of crossing the competencies of pharmacists*, *nurses*, *and physicians*. *This should be clearly defined”*

P8

“*Weakness nowadays is the lack of continuous training in PC and NP for nurses*”

P12

“It would be necessary to expand basic training and then also specialization […] but the most important is continuous training”

P20

“*I wouldn’t want to lose that multidisciplinary dynamic*”

P21

*“The lack of knowledge about drugs decrease professionals’ confidence*. *It is a threat that the nurses do not adapt to the protocols due to their lack of training”*

P23

“*The only weakness is that there’s not enough knowledge about drugs*, *honestly*”

P24

### Connecting category 2: Interprofessional collaboration & communication

We observed consensus positions throughout all interviews about the idea of working as a team towards maximizing patients’ benefits and positive populations’ health outcomes with relation to PC. They considered this to be the best way to improve the quality of healthcare. Interviewees emphasized on two main concepts to achieve this: collaboration and communication. Also the idea of a higher-level NP training within PC training for Nurses emerged. There were two models of multidisciplinary PC suggested and defined along the interviews that contributed to shape our comprehensive model:

- “In place” or “face-to-face” model: the different professionals attend the patient simultaneously and therefore the treatment is adapted and individualized at that moment, and there are no problems regarding drugs’ conciliation or communication among professionals and towards patients and families:
  *“Real multidisciplinary teams are what we need*. *For instance*, *the pharmacist would be there while we visit the patient”*
  P14
  *“One team made up of the three who attend the patient simultaneously […]*. *It is necessary to change the model”*
  P19
- “Communication flowing” model: professionals see the patient independently though they are in constant communication to detect any problem regarding drug interaction, or at a consulting level.
  “*An internal communication either via email or reports written in pigeonholes*”
  P16
  “*Coordinated transition to other services would work well*”
  P18
  “*Use of computerised programs to communicate…*”
  P20
  *“Communication should be flowing from the beginning and be understandable*. *And I think we can all contribute to it”*
  P22
  “*Prescribing of pharmaceutical products that can interact together would pop up an alert…the pharmacist identifies such situations and contacts the professional by identifying any possible interaction or any other flaw that might be present*”
  P24

## Discussion

This paper highlights the prodromes of the Spanish Nursing journey towards regulating PC and specifically NP as a key Nursing role according to other health professionals. Lessons from the past and from other countries’ experience could be drawn upon in order to better utilise the existing well-educated nursing workforce in Spain. However, in our study we have prioritised the real perception and expectancy of those stakeholders involved. Our study provides evidence that supports the idea of increasing Nursing training in pharmacology and PC and its periodically updating to reach an optimal level of knowledge to make NP possible. Two studies from the United Kingdom and the USA, where NP has been functioning for years show the importance of pharmacological training for the nurses and its implications. They demonstrated that an in-depth knowledge of pharmacology increases the confidence of health professionals [4] and increases the safety of drug choice and administration [22]. Regarding NP in Spain, similar results for collaborative and autonomous or independent competencies were obtained in a Catalonian study. The NP is linked to the nursing process and contributes effectively to the management of healthcare [23]. Contrasting the information with other countries, in the United Kingdom nurses can prescribe and they have demonstrated that training is essential, as well as the preservation of the role of nurses in care, thus not focusing all their efforts on prescribing drugs but on prescribing care [24]. Furthermore, a type of communication via one unified application for all the health professionals may improve the control and follow-up of pharmacological therapy [25]. Extrapolating these ideas to the current practice within the Spanish healthcare system may be strategic for the future. A collaborative framework based on consensus should be standardised for all healthcare environments and common to all autonomous communities. Our comprehensive model allows the understanding of the phenomenon in Spain by connecting the current PC Nursing responsibilities and tasks nowadays with the PC Nursing Ideal role through Nurse Prescribing identification of benefits and drawbacks and Interprofessional collaboration and communication.

### Limitations

The context of this qualitative study is the province of Alicante. We have chosen the most representative settings and selected in each of them those professionals who could contribute the most according to the type of sampling, the inclusion criteria and their representativeness. However, in Spain different responses from the different autonomous communities may have provided other perceptions and expectancies due to the distinct implementations of the same Royal Decree. Yet, we are convinced that the Spanish Regulation about Nurse Prescribing in the different autonomous communities (17 ones in total) may have different implementation but the same core values.

In relation with participants, some healthcare workers who are currently part of the settings selected were excluded from the interviews such as: nurse-assistants, cleaning staff, paid non-professional caregivers, administrative staff, health policymakers, etc. They may also have an opinion regarding pharmaceutical care, but in this study, we aimed to include only those considered as health professionals by the Spanish Ministry of Health Law.

## Conclusions

Even though PC and specifically NP can be conceived as a professional ‘problem construction’ nowadays in Spain, the professionals’ perceptions and expectancies explored in our study pointed out a positive future. As part of DeMoPhaC Project our study reflects that Spanish health professionals recognize nurses as the closest professionals to patients and families and directly responsible for their care. They are clearly identified by the rest of professional groups as the main professional guiding patient care including PC. NP came out as an important strategic part of PC even though consensus has not been reached by Spanish health professionals about the type of NP to be implemented. However, it is imperative to improve the quality of care regarding pharmaceutical aspects for patients in Spain by unifying the specific roles, determining the competencies, responsibilities and defining the implementation process. Professionals regard NP as an opportunity to improve the quality of patients’ cares and for the nursing profession by solving irregular situations. The adaptation of the Royal Decree Law at regional level should include some measures according to health professionals such as more training in PC at different levels of the Nursing Education. Indirectly, further training in pharmacology would increase the confidence of nurses in daily practice. The multidisciplinary working model must be linear, either simultaneously collaboration between pharmacists, nurses and physicians, or maintaining constant communication among all of them. Accordingly, all of them should work together to ensure safe administration to patients. Moreover, the implementation of NP in Spain would provide legal recognition to some related practices done nowadays and consequently would increase safety in medication management both for patients and professionals.

DeMoPhaC partnership promotes the importance of the Interprofessional Collaboration and Communication in relation with PC, assuming the nursing profession as the pivotal agent among the rest of professionals for quality and integrated person-centred care and promoting NP as a core role towards achieving efficiency in the health systems.

## Supporting information

S1 Checklist COREQ checklist. (DOCX)
