# Supplementary material for: Understanding pharmaceutical care and nurse prescribing in Spain: A grounded theory approach through healthcare professionals’ views and expectations
Source: PLoS One. 2022 Jan 24;17(1):e0260445. doi: 10.1371/journal.pone.0260445 (PMC8786147; doi:10.1371/journal.pone.0260445)
Supplement: S1 Checklist — (DOCX) [file pone.0260445.s001.docx]

**COREQ Checklist**

**Domain 1: Research team and reflexivity**

Personal Characteristics

1. Interviewer/facilitator: Which author/s conducted the interview or focus group? *(co-author 3) AM, (co-author 2) JR*

2. Credentials: What were the researcher’s credentials? *PhD (1,5,6); PhD Candidates (4,2); MNR (3)*

3. Occupation: What was their occupation at the time of the study? *Lecturers (1,5,6); Researchers (2,3,4)*

4. Gender: Was the researcher male or female? *4 Males (1,2,3,6): 2 Females (4,5)*

5. Experience and training: What experience or training did the researcher have? *Extended experience over 15 years (1,5,6); four-year experience (2,4); early-stage researcher (3)*

Relationship with participants

6. Relationship established: Was a relationship established prior to study commencement? *Yes. The Main researcher contacted with the chiefs of the selected environments/contexts.*

7. Participant knowledge of the interviewer: What did the participants know about the researcher? *Reasons for conducting the research were informed previously.*

8. Interviewer characteristics: What characteristics were reported about the interviewer/facilitator? *Background and reasons and interests in the research topic and their role within the research project.*

**Domain 2: study design**

Theoretical framework

9. Methodological orientation and Theory: What methodological orientation was stated to underpin the study? *Grounded theory*

10. Sampling: How were participants selected? *Snowball sampling*

11. Method of approach: How were participants approached? *face-to-face*

12. Sample size: How many participants were in the study? *Twenty-four*

13. Non-participation: How many people refused to participate or dropped out? Reasons? *None*

Setting

14. Setting of data collection: Where was the data collected? *At participants’ workplace*

15. Presence of non-participants: Was anyone else present besides the participants and researchers? *No*

16. Description of sample: What are the important characteristics of the sample? *demographic data (table 1 of the manuscript)*

Data collection

17. Interview guide: Were questions, prompts, guides provided by the authors? Was it pilot tested? *Guides provided by the DEMOPHAC Research Project Partnership after consensus. Also specific training about Qualitative Research for the researchers that conducted the interviews took place under the Project timeline.*

18. Repeat interviews: Were repeat interviews carried out? If yes, how many? *No*

19. Audio/visual recording: Did the research use audio or visual recording to collect the data? *Audio recording*

20. Field notes: Were field notes made during and/or after the interview or focus group? *Yes*

21. Duration What was the duration of the interviews or focus group? *Sixty minutes approximately.*

22. Data saturation: Was data saturation discussed? *Yes*

23. Transcripts returned: Were transcripts returned to participants for comment and/or correction? *Yes*

**Domain 3: analysis and findings**

Data analysis

24. Number of data coders: How many data coders coded the data? *3 experienced researchers participated in the data analysis (analysis triangulation).*

25. Description of the coding tree: Did authors provide a description of the coding tree? *Yes (table 2 of the manuscript)*

26. Derivation of themes: Were themes identified in advance or derived from the data? *Derived from data.*

27. Software: What software, if applicable, was used to manage the data? *No software was used.*

28. Participant checking: Did participants provide feedback on the findings? *Yes*

Reporting

29. Quotations presented: Were participant quotations presented to illustrate the themes / findings? Was each quotation identified? *Yes. Quotations are presented along the text including* *participant number (according to table 1)*

30. Data and findings consistent: Was there consistency between the data presented and the findings? *Yes*

31. Clarity of major themes: Were major themes clearly presented in the findings? *Yes, including one figure that represents the comprehensive model/framework to understand the phenomenon.*

32. Clarity of minor themes: Is there a description of diverse cases or discussion of minor themes? *Yes*
